# Ligand–Receptor Interactions Elucidate Sex-Specific Pathways in the Trajectory From Primordial Germ Cells to Gonia During Human Development

**DOI:** 10.3389/fcell.2021.661243

**Published:** 2021-06-18

**Authors:** Arend W. Overeem, Yolanda W. Chang, Jeroen Spruit, Celine M. Roelse, Susana M. Chuva De Sousa Lopes

**Affiliations:** ^1^Department of Anatomy and Embryology, Leiden University Medical Centre, Leiden, Netherlands; ^2^Ghent-Fertility and Stem Cell Team (G-FAST), Department of Reproductive Medicine, Ghent University Hospital, Ghent, Belgium

**Keywords:** development, human fetal gonads, single-cell transcriptomics, signaling pathways, sex-specific, gametogenesis, primordial germ cell (PGC), gonia

## Abstract

The human germ cell lineage originates from primordial germ cells (PGCs), which are specified at approximately the third week of development. Our understanding of the signaling pathways that control this event has significantly increased in recent years and that has enabled the generation of PGC-like cells (PGCLCs) from pluripotent stem cells *in vitro*. However, the signaling pathways that drive the transition of PGCs into gonia (prospermatogonia in males or premeiotic oogonia in females) remain unclear, and we are presently unable to mimic this step *in vitro* in the absence of gonadal tissue. Therefore, we have analyzed single-cell transcriptomics data of human fetal gonads to map the molecular interactions during the sex-specific transition from PGCs to gonia. The CellPhoneDB algorithm was used to identify significant ligand–receptor interactions between germ cells and their sex-specific neighboring gonadal somatic cells, focusing on four major signaling pathways WNT, NOTCH, TGFβ/BMP, and receptor tyrosine kinases (RTK). Subsequently, the expression and intracellular localization of key effectors for these pathways were validated in human fetal gonads by immunostaining. This approach provided a systematic analysis of the signaling environment in developing human gonads and revealed sex-specific signaling pathways during human premeiotic germ cell development. This work serves as a foundation to understand the transition from PGCs to premeiotic oogonia or prospermatogonia and identifies sex-specific signaling pathways that are of interest in the step-by-step reconstitution of human gametogenesis *in vitro.*

## Introduction

Understanding the germ cell life cycle requires identification of the key signaling events that decide and regulate each step of germ cell fate. This knowledge will be essential to recapitulate gametogenesis *in vitro* in humans, which is expected to be a powerful tool to study human development and generate treatments for infertility. The first step of gametogenesis in mammals is the specification of primordial germ cells (PGCs). Work in mouse embryos has identified *Prdm1* (or *Blimp1*), *Tfap2c*, and *Prdm14* as essential regulators of PGC specification, driven by BMP signaling from the extraembryonic tissues and WNT signaling from the posterior epiblast, leading to lineage restriction at 7.2 days post fertilization (dpf) (Li et al., 2020). In humans, the signaling pathways involved in PGC specification *in vivo* remain challenging to investigate; however, work on human preimplantation blastocysts cultured until day 14 dpf suggests that PGC specification may occur around 12 dpf (Chen et al., 2019; Popovic et al., 2019) and may as well be regulated by BMP and WNT signaling (Tang et al., 2016). In both humans and mice, PGCs migrate to the future gonadal ridges, where they settle as the gonads undergo sex determination. In the gonads, PGCs differentiate into gonia (GONs), prospermatogonia (SGON) in males or oogonia (OGON) in females, characterized by the upregulation of DDX4 and DAZL and downregulation of pluripotency markers such as POU5F1 and NANOG (Li et al., 2020). In males, SGONs arrest as prospermatogonia until birth, and in females, the OGONs enter meiotic prophase I and arrest in the diplotene stage (dictyate) in primordial follicles.

The formation of PGC-like cells (PGCLCs) from human and mouse pluripotent stem cells (PSCs) has been successfully recapitulated (Irie et al., 2015; Kotaro Sasaki et al., 2015; Kobayashi et al., 2017). However, the directed differentiation of PGCLCs to premeiotic (POU5F1^–^/DDX4^+^) GON-like cells has only been robustly achieved using mouse female PSCs (Miyauchi et al., 2017). In the case of mouse male PSCs and human male and female PSCs, differentiation from PSCs to (POU5F1^–^/DDX4^+^) GON-like cells has been reported, but requires a necessary step of co-culture with supporting gonadal somatic cells in the form of reconstituted ovaries or testes (Hayashi et al., 2011; Zhou et al., 2016; Yamashiro et al., 2018; Hwang et al., 2020). As such, the exact differentiation cues determining the step from PGCs to premeiotic (POU5F1^–^/DDX4^+^) SGONs and OGONs remain unclear, especially in humans where working *in vivo* remains particularly challenging. To determine the correct culture conditions for the differentiation step between human PGCs and premeiotic GONs, it is important not only to first understand the developmental trajectories of human germ cells *in vivo*, between those two developmental stages, but also to characterize the surrounding signaling environment, which provides sex-specific developmental cues.

Recent developments in single-cell transcriptomics have facilitated the generation of large online-available single-cell datasets of different organs and tissues, contributing tremendously to the cellular characterization and even the discovery of novel cell types in the human body during health and disease (Grün et al., 2015; Birnbaum, 2018; Anaparthy et al., 2019). Moreover, the ongoing development of novel freely available computational tools, particularly focusing on the analysis of transcriptional networks (Fiers et al., 2018) and receptor–ligand interactions (Armingol et al., 2020), is revolutionizing the way we optimize and validate (using machine-learning algorithms) differentiation protocols. Making use of the most comprehensive online-available single-cell RNA-sequencing (scRNA-seq) dataset of human fetal gonadal tissue (Li et al., 2017), the CellphoneDB algorithm (Efremova et al., 2020) was used to map possible ligand–receptor interactions between germ cells and surrounding gonadal somatic cells. We selected interactions representing four major developmental signaling pathways [receptor tyrosine kinases (RTK), WNT, TGFβ/BMP, and NOTCH] and have provided the cellular localization of effectors of those signaling pathways in male and female human gonads of first and second trimester. Surprisingly, the systematic analysis of these four signaling pathways suggests that the transition from PGCs to premeiotic (POU5F1^–^/DDX4^+^) GONs in humans involves cytokines, such as KITL, but seems to be regulated by sex-specific signals, in particular high levels of BMPs and canonical WNT in females, contrasting to low levels of BMPs and FGF9 together with IGF1 and activin A (ActA) in males.

## Materials and Methods

### Ethical Permission to Use Human Material

The human fetal material used in this work was obtained from elective abortions (without medical indication) with signed informed consent. This work described here was approved by the Medical Ethical Committee of Leiden University Medical Centre (P08.087).

### Collection of Human Fetal Tissue and Sex Genotyping

Human gonads (and intestine) were isolated and washed in cold 0.9% NaCl (Fresenius Kabi, Bad Homburg, Germany). Next, they were fixed in 4% paraformaldehyde (PFA, Sigma-Aldrich, St. Louis, MO, United States) under mild shaking at 4°C overnight. The gonads were washed in phosphate-buffered saline (PBS), transferred to 70% ethanol, washed several times, and stored in 70% ethanol at 4°C before paraffin embedding. When necessary, the sex was determined by genomic PCR for Amelogenin (AMELX/AMELY), which distinguishes the X and Y chromosomes by amplicon size (977 and 790 bp, respectively), as described previously (Heeren et al., 2015).

### Tissue Culture

HEK293T cells were cultured in DMEM/F12 (Thermo Fisher Scientific, Waltham, MA, United States) with 10% fetal calf serum (FCS) (Sigma-Aldrich, St. Louis, MO, United States) and 50 U/ml penicillin-streptomycin (Thermo Fisher Scientific, Waltham, MA, United States). The cells were seeded on coverslips coated with 0.1% poly-l-lysine solution (Sigma-Aldrich, St. Louis, MO, United States) for 30 min and were either cultured with culture medium (10% FCS) or serum starved (0% FCS) for 16 h. The cells that were serum starved overnight were cultured for 30 min in DMEM/F12 containing specific growth factors, 500 ng/ml IGF1 (R&D Systems, Minneapolis, MN, United States, 291-G1), 100 ng/ml SCF (R&D Systems, Minneapolis, MN, United States, 7466-SC), 20 ng/ml TGFβ1 (PeproTech, Rocky Hill, CT, United States, 100-21-A), 100 ng/ml FGF2 (PeproTech, Rocky Hill, CT, United States, 100-18B), 100 ng/ml EGF (R&D Systems, Minneapolis, MN, United States, 236-EG), 200 ng/ml BMP4 (R&D Systems, Minneapolis, MN, United States, 314-BP), or 100 ng/ml activin A (R&D Systems, Minneapolis, MN, United States, 338-AC). Thereafter, the cells were fixed in 4% PFA for 20 min at room temperature and used for immunofluorescent staining.

### Immunofluorescence and Imaging

Fetal gonads (and intestine) were embedded in paraffin using a Shandon Excelsior tissue processor (Thermo Fisher Scientific, Waltham, MA, United States) and sectioned (5 μm) using a RM2065 microtome (Leica Instruments, Wetzlar, Germany) onto StarFrost slides (Waldemar Knittel, Brunswick, Germany). Paraffin sections were deparaffinized in xylene and rehydrated in an ethanol dilution series ending with water. Antigen retrieval was performed in 0.01 M citric buffer (pH 6.0) for 12 min at 98°C in a TissueWave 2 Microwave (Thermo Fisher Scientific, Waltham, MA, United States). After rinsing with PBS, sections were incubated for 1 h at room temperature with blocking solution [1% bovine serum albumin (BSA, Sigma-Aldrich, St. Louis, MO, United States), 0.05% Tween-20 (Merck, Darmstadt, Germany)]. Samples were incubated with primary antibodies diluted in blocking solution overnight at 4°C, washed three times with PBS, and incubated with secondary antibodies and DAPI (Thermo Fisher Scientific, Waltham, MA, United States) diluted in blocking solution for 1 h at room temperature. Slides were washed three times with PBS and mounted with coverslips using ProLong Gold (Thermo Fisher Scientific, Waltham, MA, United States).

Cells on coverslips were fixed in 4% PFA for 20 min, washed three times with PBS and permeabilized with 0.2% Triton X-100 (Sigma-Aldrich, St. Louis, MO, United States) in PBS, and washed three times with 0.05% Tween-20 (Sigma-Aldrich, St. Louis, MO, United States) in PBS. Next, the cells were incubated for 1 h at room temperature with blocking solution, followed by incubation with primary antibodies diluted in blocking solution overnight at 4°C, washed three times with PBS, and incubated with secondary antibodies and DAPI diluted in blocking solution for 1 h at room temperature. Finally, cells were washed with PBS and the coverslips were mounted on StarFrost slides (Waldemar Knittel Brunswick, Germany) using ProLong Gold.

A list of the used antibodies can be found in Supplementary Table 1. Slides were analyzed on a Leica SP8 confocal microscope, and grayscale images were combined and edited (adjustment of brightness/contrast) in Adobe Photoshop.

### Analysis of RNA Sequencing Data

The count table in unique molecular identifiers (UMI) was made available by Li et al. (2017)^1^. The dataset was loaded in R (version 4.0.2) as a Seurat object, and cells expressing less than 250 genes, as well as genes expressed in less than 10 cells, were filtered from the dataset. Functions specified here belong to Seurat (version 3.1.4) (Stuart et al., 2019) unless noted otherwise. The dataset was divided according to sex and separately normalized (log-normalization as per NormalizeData function with a scale factor of 10,000). The variable genes in the male and female datasets were determined (FindVariableFeatures), followed by scaling (ScaleData), and analyzed using the SCORE algorithm (RSCORE package version 0.1.0) (Dong et al., 2019). A principal component analysis (PCA) was performed (RunPCA), visualized using uniform manifold approximation and projection (UMAP) (RunUMAP, PCA dimensions 1:40 for male and 1:10 for female), and clustered (FindNeighbors and FindClusters). Single gene expression UMAP and violin plots were generated using the FeaturePlot and VlnPlot functions, respectively.

For analysis of differentially expressed genes (DEG), the expression levels were compared (pair-wise) using the FindMarkers function. A full list of DEGs (Supplementary Table 2) and pathway-specific DEGs (Supplementary Table 3) is included. Volcano plots were generated using the EnhancedVolcano package (version 1.8.0) (Blighe et al., 2020), and heatmap plots (dendrograms obtained by complete linkage clustering) were generated using pheatmap package (version 1.0.12) (Kolde, 2019). To obtain DEGs of particular signaling pathways (RTK, WNT, TGFβ/BMP, and NOTCH), the total DEG list of these pathways was filtered as defined by the Kyoto Encyclopedia of Genes and Genomes (KEGG)^2^ (KEGG IDs: 04010, 04310, 04330, 04350, and 04151). RTKs do not have a separate KEGG pathway but instead are part of several. Two of these, MAPK- (04010) and PI3K-Akt (04151), encompass the major effectors of downstream RTK signaling and were used for analysis of RTK-associated signaling. Schematic signaling models were adapted and simplified from the five KEGG pathway maps and manually overlaid with color gradients indicating average log(fold change).

### Receptor–Ligand Interaction Analysis Using CellphoneDB

For receptor–ligand expression analysis, count data was extracted from R-based Seurat objects and used as input for the CellphoneDB algorithm (version 2.1.2) in Python 3.8 (Efremova et al., 2020). Using of sex-specific clusters of interest, we filtered for receptor–ligand interactions with *P* < 0.1 and for which the component genes (UMI > 0) were expressed in >30% cells in the clusters of interest. Note that in CellphoneDB, the database of available receptor–ligand interactions is limited to a fixed set of experimentally validated interactions; hence, not validated interactions are absent. From the obtained subset of receptor–ligand interactions between the clusters of interest, we selected receptor–ligand pairs involved in the RTK, WNT, TGFβ/BMP, and NOTCH signaling pathways. For this, the following key (partial) gene symbols to filter the results were used: WNT signaling: WNT, FZD, RSPO, LGR, LRP5, and LRP6; TGFβ/BMP signaling: ACVR, BMP, INHBA, INHBB, AMH, MIS, and TGF; NOTCH signaling: NOTCH; and RTK signaling: CSF1R, EGFR, EPHA2, ERBB2, ERBB3, ERBB4, FGFR, FGFR2, FGFR3, FGFR4, FLT1, FLT3, FLT4, IGF1R, INSR, KDR, KIT, MET, NGFR, NTRK1, NTRK2, PDGFRA, PDGFRB, and TEK. The selection of RTK genes was based on the definition of RTKs in the MAPK- and PI3-Akt signaling KEGG pathways (IDs: 04010 and 04151). The results were visualized using the dot_plot function of CellphoneDB.

### Statistics

For receptor–ligand analysis, statistical data was generated by the CellphoneDB algorithm, which uses empirical shuffling to determine whether ligand–receptor pairs show cell cluster specificity (see Efremova et al., 2020). For DEG analysis, results were tested with Wilcoxon rank-sum test and adjusted with a Bonferroni correction (referred to as adjusted *p*-value in figures).

## Results

### Identification of Different Cell Types in the Human Fetal Gonads

**FIGURE 1:**
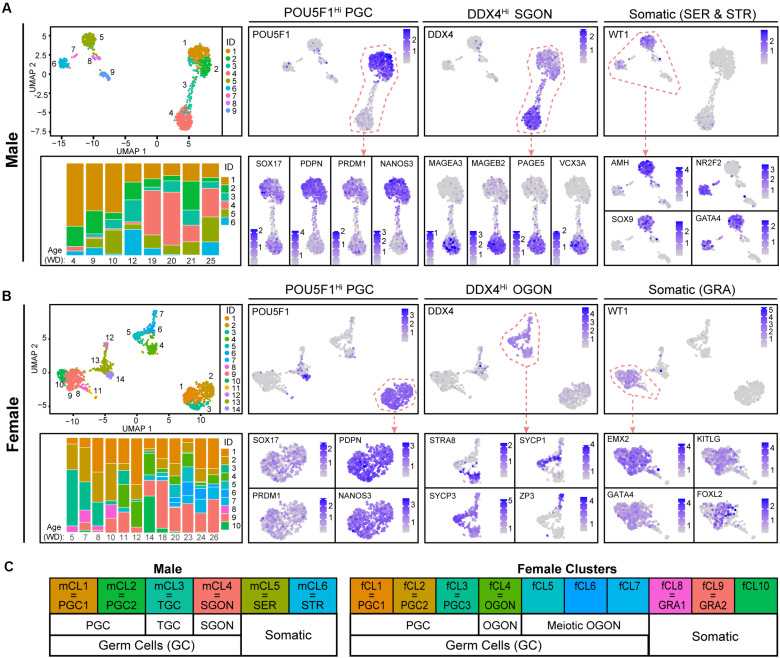
Cell clustering in human fetal male and female gonads. **(A,B)** Uniform manifold approximation and projection (UMAP) visualization of cells from the human fetal testis **(A)** or ovary **(B)** showing cluster identification (ID) (top left panel) and the expression of genes of interest [log-normalized unique molecular identifiers (UMI)] for cell type identification (right side). Bottom left panels show stacked bar plots depicting relative distribution of cells in each cluster per age (weeks of development, WD) in the dataset. **(C)** Schematic overview of the cell types assigned to the different clusters.

We analyzed publicly available scRNA-seq data from developing human fetal male and female gonads of 4–26 weeks of development (WD), corresponding to 6–28 weeks of gestation (WG) (Li et al., 2017). For this, the dataset was first separated by sex, followed by cell clustering on each set individually and visualization by UMAP. In the male set (Figure 1A), clusters (mCL)1–4 represented germ cells, with cells in mCL1 and mCL2 corresponding to PGCs (*POU5F1*, *SOX17*, *PDPN*, *PRDM1*, and *NANOS3*) and mCL4 to SGONs, expressing high levels of *DDX4*, as well as male-specific germ cell markers such as *MAGEA3*, *MAGEB2*, *PAGE5*, and *VCX3* and no *POU5F1* (Lahn and Page, 2000; Simpson et al., 2005; Lee and Potts, 2017). mCL3 represented a transitory state between PGCs and SGONs, which we named transitory germ cells (TGCs). These cells were characterized by downregulation of PGC markers and low DDX4 expression. The male somatic clusters mCL5 and mCL6 corresponded to Sertoli cells (SER) (*WT1*, *GATA4*, *AMH*, and *SOX9*) and stromal cells (STR) (*NR2F2* and *GATA4*), respectively (Figure 1A and Supplementary Figure 1A). In the female set (Figure 1B), we identified fCL1–7 as germ cells. Female PGCs (*POU5F1*, *PDPN*, *PRDM1*, and *NANOS3*) were in fCL1–3. fCL4–7 contained more mature *POU5F1*^–^*/DDX4*^+^ OGONs, which sub-clustered into premeiotic retinoic acid (RA)-responsive (fCL4, expressing *STRA8)*, meiotic (fCL5 and fCL6, expressing both SYCP1 and SYCP3), and dictyate (fCL7, expressing *ZP3*) OGONs. In contrast to males, females lacked a cluster transiting from PGCs to GONs. Based on expression of *WT1*, *FOXL2*, *KITLG*, and *GATA4*, fCL8–10 were identified as (pre-)granulosa cells (GRA) (Figure 1B and Supplementary Figure 1A). This classification is largely in agreement with that proposed previously (Li et al., 2017). A schematic overview of terminology is provided in Figure 1C.

**FIGURE 2:**
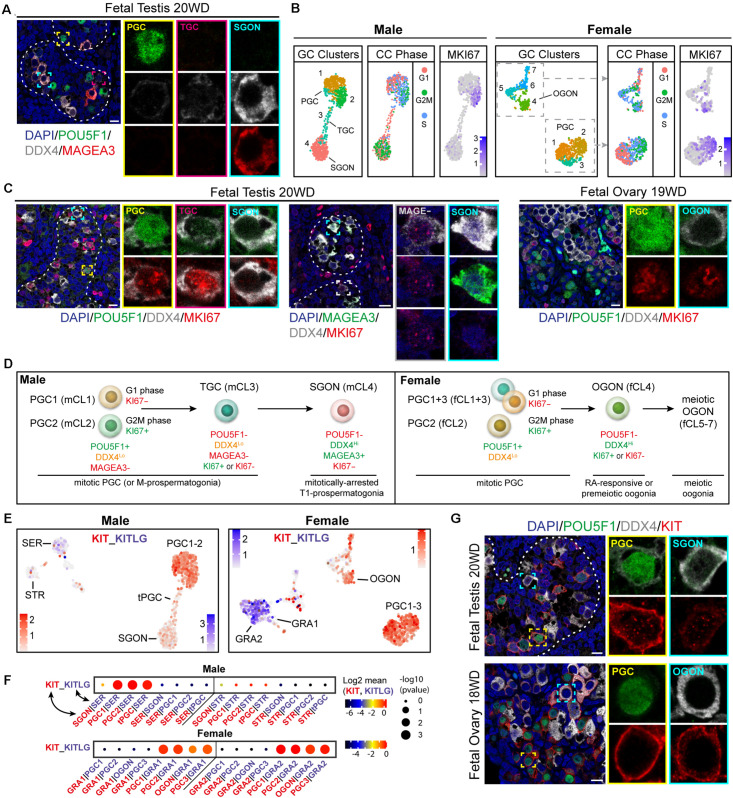
Characterization of germ cell proliferative state and validation of the receptor–ligand analysis method. **(A)** Immunofluorescence of MAGEA3, POU5F1, and DDX4 in fetal testis (18 WD), revealing three germ cell types: POU5F1^+^/DDX4^low^/MAGEA4− primordial germ cells (PGCs), POU5F1−/DDX4^high^/MAGEA4^+^ SGON, and POU5F1−/DDX4^low^/MAGEA4− transitory germ cells (TGCs). **(B)** UMAP visualization of isolated male (left panels) and female (right panels) germ cell (GC) clusters, showing cluster identification, estimated cell cycle (CC) phase, and MKI67 expression. Both male and female PGC show variable CC state and expression of MKI67, reflecting proliferation. However, gonia (GONs) differ between male and female, with MKI67 being expressed in oogonia (OGONs), but not in prospermatogonia (SGONs). **(C)** Immunofluorescence of MKI67 in fetal testis (18 WD) and ovary (19 WD). A subset of PGCs (POU5F1+) and TGCs (POU5F1−/DDX4^low^) shows positive MKI67 staining, whereas SGONs (left panel: POU5F1−/DDX4^*Hi*^, middle panel: MAGEA3^+^/DDX4^*Hi*^) were never MKI67 positive. In fetal ovary, both PGC and premeiotic OGONs MKI67^+^ subsets are present. **(D)** Overview of identified germ cell subtypes, the markers used for their identification, and their proliferative state. **(E)** UMAP plots of male and female sets showing expression levels of *KIT* (in red) and *KITLG* (in blue). KIT is expressed in PGC clusters and premeiotic OGONs, while KITLG is expressed by GRA1–2 and Sertoli cells (SER). **(F)** CellphoneDB analysis of KIT–KITLG interaction between different clusters in male (top) and female (bottom) fetal gonads. Depicted are −log_10_ *p*-values (circle size) and log_2_ means (circle color) for the KIT–KITLG interacting pair, for selected pairwise cluster combinations. In the ovary, a significant KIT–KITLG interaction is observed from GRA to PGC and OGON clusters, while in males, a significant interaction is observed from SER to PGC clusters only. **(G)** Immunofluorescence of KIT with POU5F1 and DDX4, in PGCs (POU5F1^+^) and GON (POU5F1-) in fetal testis (20 WD) and ovary (18 WD). Consistent with scRNA-seq data, KIT is expressed by PGCs and OGONs, but not SGONs. Yellow boxes indicate zoom-in of a PGC, and cyan boxes indicate zoom-in of a GON. Scale bar is 10 μm.

For SGONs, the prefixes multiplying (M)-, transitional-1 (T1), and T2 are conventionally used to differentiate developmental states, based on proliferation (Hilscher et al., 1974; Culty, 2013). Moreover, three male germ cell populations were previously reported in the testis, based on the expression of POU5F1, KIT, and MAGEA3/A4 (Gaskell et al., 2004). In agreement, we observed three germ cell populations: POU5F1^+^/DDX4^low^/MAGEA4^–^ PGCs, POU5F1^–^/DDX4^high^/MAGEA4^+^ SGONs, and POU5F1^–^/DDX4^low^/MAGEA4^–^ cells, likely TGCs (Figure 2A and Supplementary Figure 1B). TGCs were also negative for NANOG and showed low expression of SOX17 (Supplementary Figure 1C). In addition, mCL1 and mCL2 differed in cell cycle state, with mCL2 being MKI67^+^ (Figure 2B). By contrast, SGONs (mCL4) were MKI67 negative. Immunofluorescence revealed the presence of MKI67^+^ PGCs (POU5F1^+^) and MKI67^+^ TGCs (POU5F1^–^/DDX4^low^) (Figure 2C and Supplementary 1D). MAGEA3 and MKI67 were mutually exclusive, confirming that MAGEA3 marks mitotically inactive SGONs (Supplementary Figure 1D). A similar distinction was not observed in female germ cells, where both PGCs and premeiotic OGONs (variably) express MKI67 (Figure 2C). A schematic overview of the identified germ cells, relevant nomenclature, and identifying markers is provided in Figure 2D.

### Validation of KIT–KITL Ligand–Receptor Interactions Using CellphoneDB

We first used the CellphoneDB algorithm (Efremova et al., 2020) on a well-known validated interaction between germ cells and surrounding somatic cells and the interaction between KIT (expressed in germ cells) and KITLG (expressed in gonadal somatic cells) (Robinson et al., 2001; Gkountela et al., 2013; Li et al., 2017). KIT belongs to the family of RTKs, a large class of proteins that activate downstream signaling cascades, including those based on MAPK- and PI3K-Akt signaling. Indeed, *KIT* is highly expressed in female and male PGCs and OGONs (fCl4), whereas its ligand *KITLG* is expressed in gonadal somatic cells (GRA1+ 2 and SER) (Figure 2E). The CellphoneDB algorithm identified this interaction as highly significant in the case of PGC-GRA1 + 2 and OGON-GRA1 + 2 in females and PGC-SER in males (Figure 2F). Interestingly, *KIT–KITLG* signaling between SGONs and SER was not significant (Figure 2F). To validate that, we confirmed that KIT was expressed in female and male PGCs and OGONs, but strongly downregulated in SGONs (Figure 2G).

### RTK Signaling Ligand–Receptor Interactions Between Germ Cells and Gonadal Somatic Cells

**FIGURE 3:**
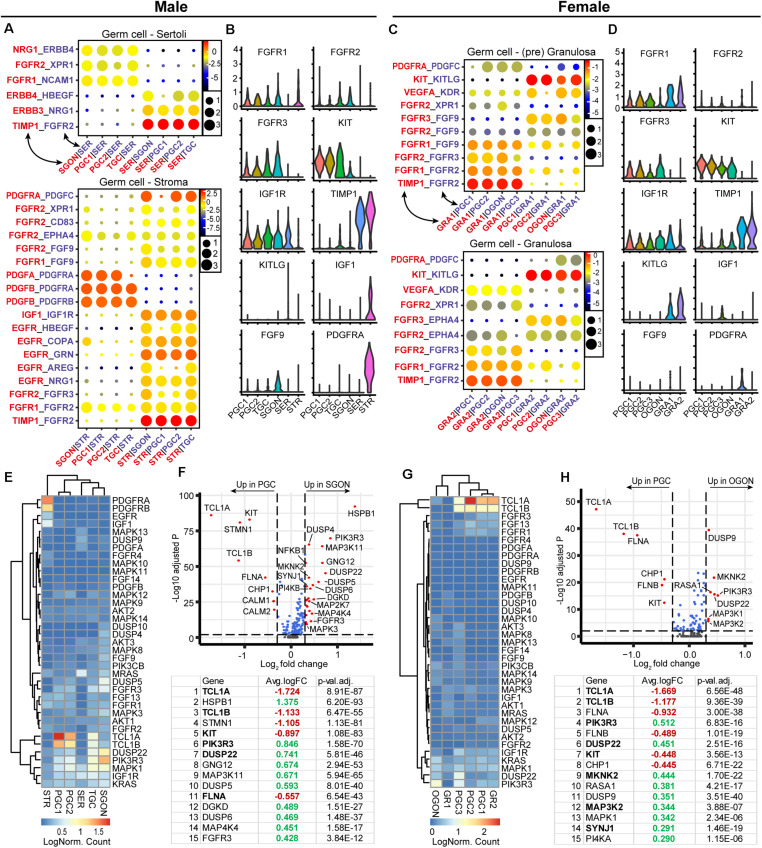
Expression of receptor tyrosine kinases (RTK) ligand–receptor pairs and downstream pathway effectors in fetal gonads. **(A)** CellphoneDB analysis of RTK interactions between different clusters in male fetal gonads. Depicted are −log_10_ *p*-values (circle size) and log_2_ means (circle color) for the interacting pairs, for selected pairwise cluster combinations. **(B)** Violin plots of selected RTK ligands and receptors in male germ and somatic clusters. **(C)** CellphoneDB analysis of RTK interactions between different clusters in female fetal gonads. **(D)** Violin plots of selected RTK ligands and receptors in female germ and somatic clusters. **(E)** Heatmap showing the expression of genes of interest associated with RTK signaling pathway in male germ and somatic cell clusters. **(F)** A volcano plot showing RTK-related genes between PGC and GON clusters in male gonads. Table shows the top 15 differentially expressed genes. Genes in bold are common between male and female [see **(H)**]. **(G)** Heatmap showing the expression of genes of interest associated with RTK signaling pathway in female germ and somatic cell clusters. **(H)** A volcano plot showing RTK-related genes between PGC and GON clusters in female gonads. Table shows the top 15 differentially expressed genes.

In addition to KIT–KITL, we investigated the presence of other significant ligand–receptor interactions between germ cells and surrounding somatic cells belonging to the RTK signaling pathway, involving cytokines such as PDGF, VEGF, IGF, and FGF (Figure 3). Interestingly, many of these interactions represented signaling from the germ cells to somatic cells (Figures 3A–D). For example, STR expressed *EGFR* and *PDGFR*, whereas their binding partners *GRN* and *PDGF* were expressed by germ cells (Figure 3A). The most significant interactions regarding signaling from somatic cells to germ cells involved *FGFR*s, *IGF1R*, and *KIT* (Figures 3A–D). Germ cells (PGCs and GONs) of both sexes expressed *IGF1R*, but its ligand *IGF1* was highly expressed only by STR (Figures 3B,D), suggesting a male-specific effect. Moreover, several *FGFR* were expressed in germ cells of both sexes, suggesting an ability to activate FGF signaling (Figures 3B,D), but surprisingly, *FGF9* was not produced by SER, as has been described in mice (Colvin et al., 2001; Kim et al., 2006), but instead by SGONs in humans (Figures 3B,D). Somatic cells of both sexes showed high expression of *TIMP1*, a secreted inhibitor of metalloproteinases with signaling properties associated with anti-apoptosis and cell growth (Jackson et al., 2017). Although the interaction between TIMP1 and FGFR2 is validated, the functional consequences of the interaction remains unclear (Huttlin et al., 2017).

Downstream of RTKs, we observed the upregulation of many key pathway effectors in GONs of both sexes: RAS (*KRAS/MRAS*), ERK (*MAPK1/3*), JNK (*MAPK8/9/10*), and PI3K (*PIK3CB/PIK3R3*) (Figures 3E–H), suggesting that the machinery downstream of RTK is in place in GONs. However, we also observed that genes involved in the negative regulation of the RTK were also differentially expressed in GONs: MAPK phosphatases (MKPs), such as *DUSP9* and *DUSP4/5/10*, were significantly upregulated in OGONs and SGONs, respectively, and *TCL1A* and *TCL1B* (which potentiates PI3K-Akt signaling) were strongly downregulated in GONs in general (Figures 3E–H).

**FIGURE 4:**
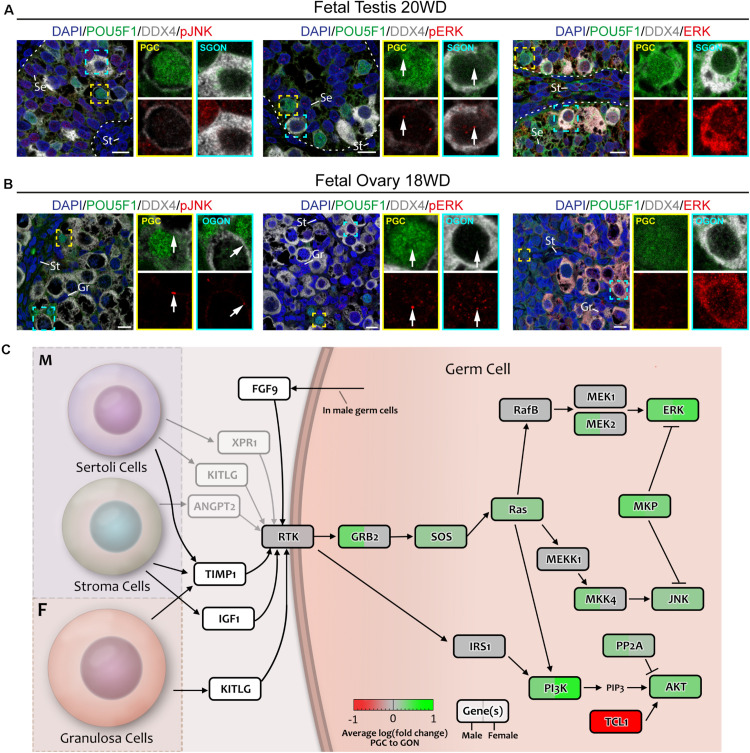
Analysis of MAPK signaling downstream of RTKs in fetal gonads by immunostaining for key (phosphorylated) pathway effectors. **(A,B)** Immunofluorescence of phosphorylated (p)-JNK, pERK, or ERK with DDX4 and POU5F1 in fetal testis of 20 weeks development (WD) **(A)** and fetal ovary of 18 WD **(B)**. No nuclear pJNK is observed in male **(A)** or female **(B)** germ cells. Total ERK levels are greatly increased in GONs of both sexes, but this is not accompanied by a corresponding increase in nuclear pERK. Yellow boxes indicate zoom-in of a PGC (POU5F1^+^), and cyan boxes indicate zoom-in of a GON (POU5F1^–^). Se, St, and Gr annotate Sertoli, stromal, and granulosa cells, respectively. Scale bar indicates 10 μm. **(C)** A schematic model of RTK signaling axes in germ cells, representing a combination of the results from CellphoneDB analysis and differentially expressed gene analysis. Genes upregulated in GONs are depicted in green, while those upregulated in PGCs are depicted in red. Gray arrows indicate interactions with low mean expression of the ligand.

To confirm the activity status of RTK pathway, we investigated the nuclear translocation of several phosphorylated (p) MAPKs, a critical event in the activation of RTK signaling. Nuclear pJNK (pMAPK8/9/10) was absent from all germ cells but was observed in male somatic cells (Figures 4A,B; control in Supplementary Figure 3A). Of note, some female germ cells (POU5F1^+^ PGCs and POU5F1^–^ OGONs) showed pJNK localized to a spot in the plasma membrane (Figure 4B). Nuclear phosphorylated-p38 MAPK signal was low in germ cells and mostly observed in somatic cells (Supplementary Figure 2A; control in Supplementary Figure 3A). However, in agreement with the transcriptomics data, total ERK (MAPK1/2) was highly expressed by GONs, particularly in the cytoplasm (Figures 4A,B and Supplementary Figure 2B), whereas punctate nuclear pERK (pMAPK1/2) was observed in somatic and germ cells of both sexes (Figures 4A,B; control in Supplementary Figure 3A), suggesting some degree of RTK activation in all cells (Figure 4C). The functional significance of the strong cytoplasmic accumulation of total ERK (MAPK1/2) in GONs remains to be further elucidated but could prepare GONs for meiotic entry (after RA signaling) (Kim et al., 2019). In agreement, leptotene/zygotene OGONs show some degree of nuclear ERK/pERK (Figure 4B).

### WNT Signaling Ligand–Receptor Interactions Between Germ Cells and Gonadal Somatic Cells

**FIGURE 5:**
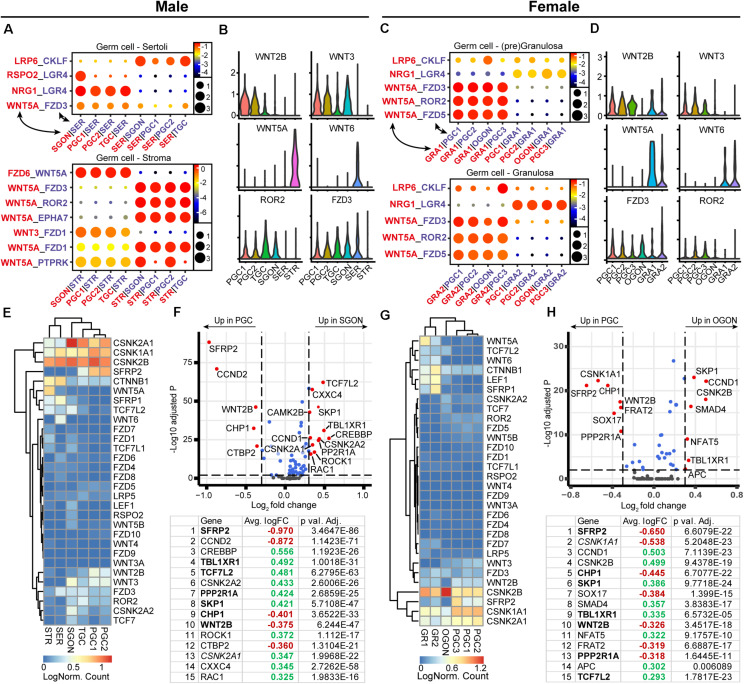
Expression of WNT ligand–receptor pairs and downstream pathway effectors in fetal gonads. **(A)** CellphoneDB analysis of WNT interactions between different clusters in male fetal gonads. Depicted are −log_10_ *p*-values (circle size) and log_2_ means (circle color) for the interacting pairs, for selected pairwise cluster combinations. **(B)** Violin plots of selected WNT-associated ligands and receptors in male germ and somatic clusters. **(C)** CellphoneDB analysis of WNT interactions between different clusters in female fetal gonads. **(D)** Violin plots of selected WNT-associated ligands and receptors in female germ and somatic clusters. **(E)** Heatmap showing the expression of genes of interest associated with WNT signaling pathway in male germ and somatic cell clusters. **(F)** A volcano plot showing WNT-related genes between PGC and GON clusters in male gonads. Table shows the top 15 differentially expressed genes. Genes in bold are common between male and female, while genes in italics show divergent regulation. **(G)** Heatmap showing the expression of genes of interest associated with WNT signaling pathway in female germ and somatic cell clusters. **(H)** A volcano plot showing WNT-related genes between PGC and GON clusters in female gonads. Table shows the top 15 differentially expressed genes. Genes in bold are common between male and female, while genes in italics show divergent regulation.

Due to a pronounced role in sex determination of the gonads (Capel, 2017), the WNT signaling pathway was next investigated in fetal gonads (Figure 5). Using CellphoneDB, we identified a significant interaction between WNT-ligand *WNT5A*, expressed by male STR and female GRA1/2, and WNT receptors *FZD3* and *ROR2*, expressed in PGCs and GONs (Figures 5A–D). As many WNT ligand–receptor interactions have not been experimentally confirmed, being therefore absent from CellphoneDB, we also studied the expression of all WNT ligands and observed that *WNT6* is expressed by somatic cells of both sexes (SER in males and GRA1/2 in females) (Figures 5B,D). Moreover, both PGCs and GONs of both sexes expressed *WNT2B* and *WNT3*, which may induce autocrine WNT signaling, although direct interactions with *FZD3* and *FZD5* have not been reported (Dijksterhuis et al., 2014).

**FIGURE 6:**
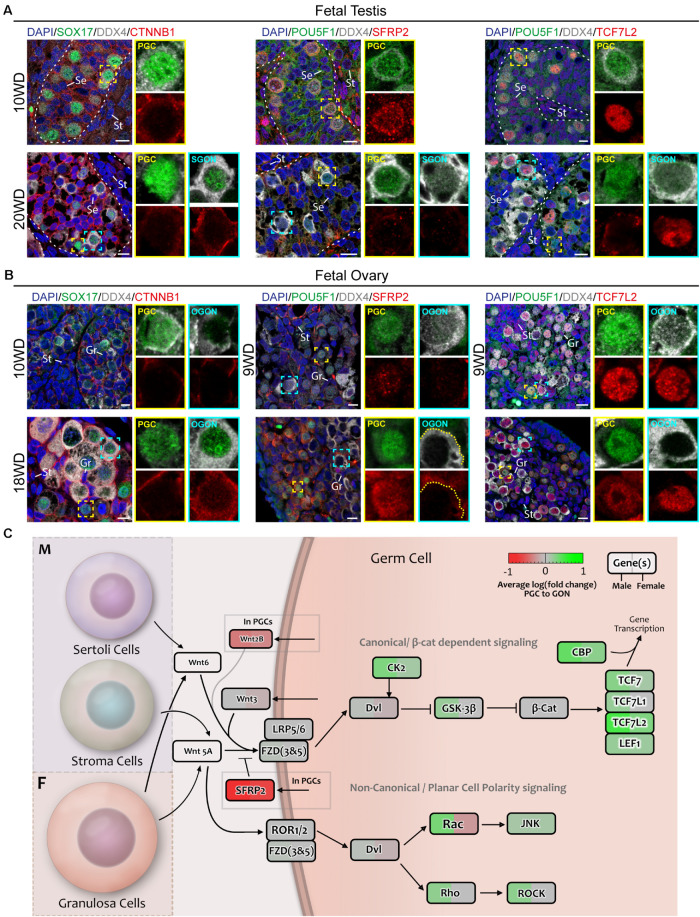
Analysis of WNT signaling effectors and downstream targets in fetal gonads by immunostaining. **(A)** Immunofluorescence of CTNNB1, SFRP2, or TCF7L2, with DDX4 and POU5F1 or SOX17 in the first trimester (10 WD, top) or second trimester (20 WD bottom) fetal testis. No nuclear CTNNB1 is observed in germ cells, suggesting that canonical WNT signaling is not active. SFRP2 expression is age dependent, being expressed in 10-WD PGCs, but not 20-WD PGCs. In 20 WD germ cells, TCF7L2 is upregulated in GONs (POU5F1−). Yellow boxes indicate zoom-in of a PGC (POU5F1^+^), and cyan boxes indicate zoom-in of a GON (POU5F1−). Se, St, and Gr annotate Sertoli, stromal, and granulosa cells, respectively. Scale bar indicates 10 μm. **(B)** Immunofluorescence of CTNNB1, SFRP2, or TCF7L2, with DDX4 and POU5F1 or SOX17 in the first trimester (9 or 10 WD, top) or second trimester (18 WD, bottom) fetal ovary. Faint staining of CTNNB1 is observed in some 18-WD GONs, suggesting (modest) activation of canonical WNT signaling. SFRP2 expression is highly upregulated in PGCs compared to GONs, especially in 18-WD ovary. TCF7L2 is expressed in both PGC and OGONs. **(C)** A schematic model of active signaling WNT axes in germ cells representing a combination of results from CellphoneDB analysis and differentially expressed gene analysis. Genes upregulated in GONs are depicted in green, while those upregulated in PGCs are depicted in red.

Next, we investigated the expression of WNT signaling pathway members between PGCs and GONs (Figures 5E–H). Casein kinase 2 (CK2) can act as a potentiator of WNT signaling, and its subunits alpha 1 and 2 (*CSNK2A1* and *CSNK2A2*) were specifically upregulated in SGONs (Figures 5E,F). In mice, *CSKN2A2* is required for spermatogenesis (Xu et al., 1999). Moreover, several members of the TCF/LEF family, such as *TCF7L2*, were upregulated in GONs of both sexes (Figures 5E,F). The TCF/LEF family plays an important role in canonical WNT signaling, allowing nuclear CTNNB1 (β-catenin) to associate with DNA and subsequently regulate transcription (Cadigan and Waterman, 2012). In addition, *SFRP2* was strongly downregulated upon transition from PGCs to GONs in box sexes (Figures 5E–H). This gene encodes a secreted FZD-related protein that is traditionally considered an inhibitor of canonical WNT signaling, but which may act as an agonist in some circumstances (van Loon et al., 2020). We confirmed the nuclear expression of TCF7L2 in most germ cells, in particular GONs (Figures 6A,B, right panels) and downregulation of SFRP2 in GONs (Figures 6A,B, middle panels), which in fetal ovary generates a gradient originating from the cortex (Supplementary Figure 2E).

To further infer on the activation of the canonical WNT signaling pathway during the transition from PGCs to GONs, we investigated the localization of CTNNB1 (β-catenin) in fetal gonads. High levels of membrane-associated CTNNB1 were present in SER and germ cells in seminiferous tubes and in GRA and germ cells in ovarian cords, but faint nuclear CTNNB1 was only detected in OGONs prior to meiotic entry (Figures 6A,B, left panels), suggesting that although the machinery for canonical WNT signaling is present in both sexes, it may be transiently active only in OGONs (Figure 6C).

### TGFβ/BMP Ligand–Receptor Interactions Between Germ Cells and Gonadal Somatic Cells

**FIGURE 7:**
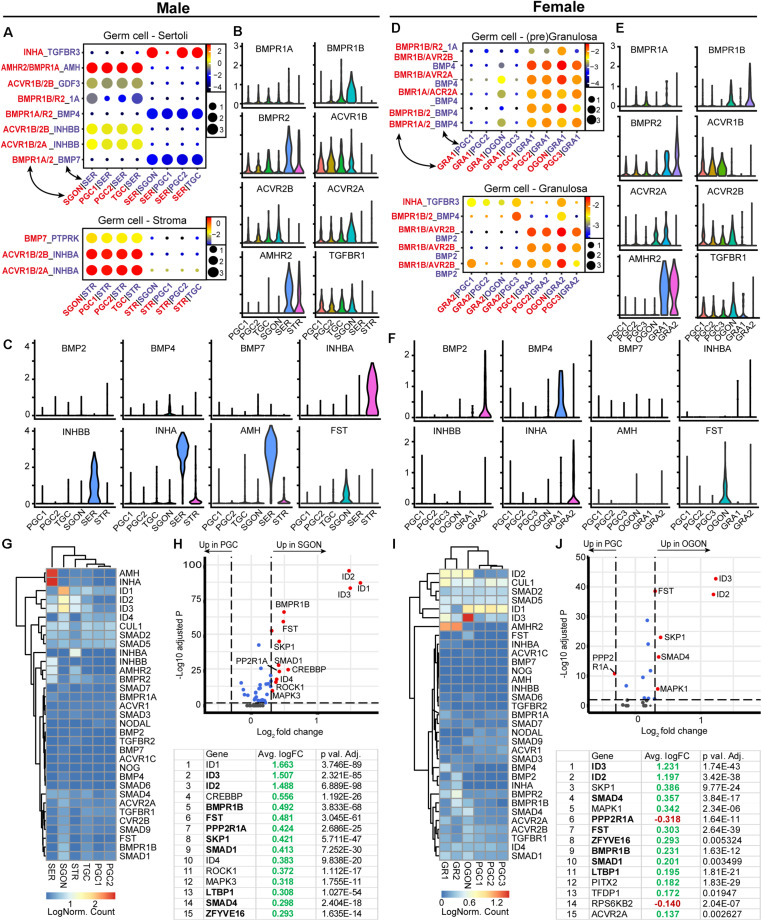
Expression of TGFβ/BMP ligand–receptor pairs and downstream pathway effectors in fetal gonads. **(A)** CellphoneDB analysis of TGFβ/BMP interactions between different clusters in male fetal gonads. Depicted are −log_10_ *p*-values (circle size) and log_2_ means (circle color) for the interacting pairs, for selected pairwise cluster combinations. **(B,C)** Violin plots of selected TGFβ/BMP-associated ligands and receptors in male germ and somatic clusters. **(D)** CellphoneDB analysis of TGFβ/BMP interactions between different clusters in female fetal gonads. **(E,F)** Violin plots of selected TGFβ/BMP-associated ligands and receptors in female germ and somatic clusters. **(G)** Heatmap of expression showing genes of interest associated with TGFβ/BMP signaling pathway in male germ and somatic cell clusters. **(H)** A volcano plot showing TGFβ/BMP-related genes between PGC and GON cluster in male gonads. Table shows the top 15 differentially expressed genes. Genes in bold are common between male and female, while genes in italics show divergent regulation. **(I)** Heatmap showing the expression of genes of interest associated with TGFβ/BMP signaling pathway in female germ and somatic cell clusters. **(J)** A volcano plot showing TGFβ/BMP-related genes between PGC and GON clusters in female gonads. Table shows the top 15 differentially expressed genes.

An analysis of TGFβ/BMP ligand–receptor interactions between germ cells and surrounding somatic gonadal cells revealed striking differences between males and females (Figure 7). CellphoneDB revealed no significant BMP interactions in males (Figures 7A–C), although low levels of *BMP4* and *BMP7* were observed in male germ cells, suggesting autocrine signaling. In females, BMP signaling in germ cells is likely to occur through *BMP2* and *BMP4*, expressed by GRA2 and GRA1, respectively (Figures 7D–F). Conversely, no inhibin genes were expressed in female gonads (Figure 7F), but *INHA* and *INHBB* were expressed by SER and *INHBA* by STR (Figure 7C). Activins and inhibins are protein dimers made of different combinations of *INHA*, *INHBA*, and *INHBB* subunits. Heterodimerization of *INHBB* and *INHA* may occur in SER, resulting in the secretion of inhibin B complex. In STR, dimerization can only occur between *INHBA*, resulting in the production of activin A, together suggesting a strong sex-specific involvement of the TGFβ/BMP signaling pathway.

The expression of TGFβ/BMP signaling pathway members revealed high expression of BMP ligand AMH (and its receptor AMHR2) specifically by SER (Figures 7C,G) and significant upregulation of several downstream effectors *SMADs* and target genes *IDs* in GONs compared with PGCs in both sexes (Figures 7G–J). The increased levels of *ID* and *SMAD1* genes in GONs suggest that BMP signaling may play an important role during the transition from PGCs to GONs. Expression levels of *SMAD2* and *SMAD3*, downstream of inhibin/activin signaling, showed low expression in both PGCs and GONs (Figures 7G,I).

**FIGURE 8:**
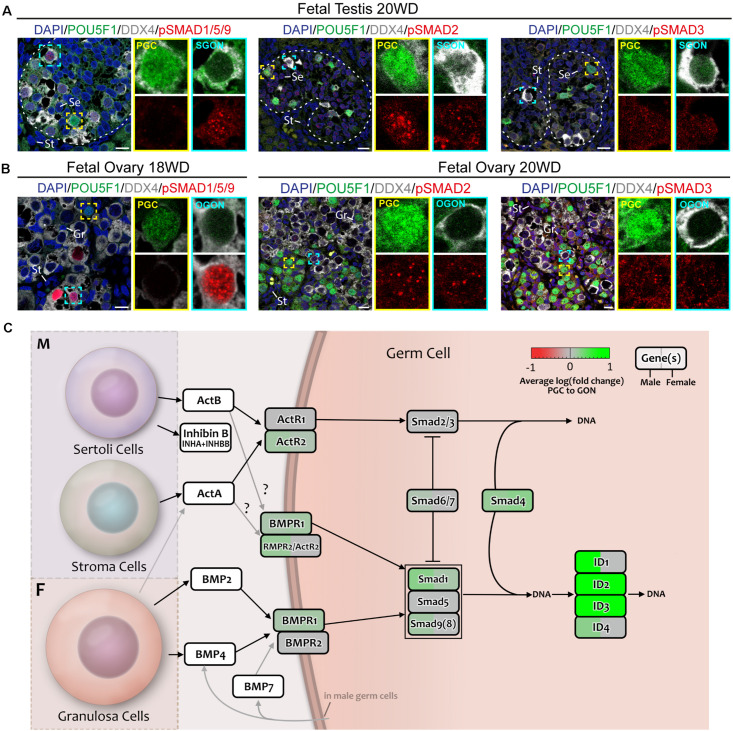
Analysis of TGFβ/BMP signaling in fetal gonads by immunostaining for key (phosphorylated) pathway effectors. **(A,B)** Immunofluorescence of phosphorylated (p)-SMAD1/5/9 and pSMAD2/3 with DDX4 and POU5F1 in fetal testis of 20 WD **(A)** and fetal ovary of 18 WD **(B)**. In the testis, pSMAD1/5/9 is localized in SGONs, whereas in the ovary, it is localized to a subset of OGONs. In contrast, pSMAD2 and 3 are present in all cell types of both the ovary and testis. Yellow boxes indicate zoom-in of a PGC, and cyan boxes indicate zoom-in of a GON. Se, St, and Gr annotate Sertoli, stromal, and granulosa cells, respectively. Scale bar indicates 10 μm. **(C)** A schematic model of active signaling BMP/TGFβ axes in germ cells representing a combination of results from CellphoneDB analysis and differentially expressed gene analysis. Genes upregulated in GONs are depicted in green, while those upregulated in PGCs are depicted in red.

A key event in the activation of TGFβ/BMP signaling pathway is the nuclear translocation of phosphorylated (p) SMAD proteins. Therefore, we examined the localization of pSMAD1/5/9 (SMAD9 is also known as SMAD8) and pSMAD2/3 by immunofluorescence in fetal gonads (Figures 8A,B; control in Supplementary Figure 3A). First, we analyzed second trimester gonads that contain both PGCs and GONs. In male 20 WD gonads, SGONs showed distinct nuclear dots of pSMAD1/5/9, while immature POU5F1^+^ PGCs did not (Figure 8A). In female 18 WD gonads, POU5F1^+^ PGCs showed no pSMAD1/5/9, but (premeiotic) OGONs showed varying levels of nuclear pSMAD1/5/9 (Figure 8A). One reason to explain the encountered heterogeneity in DDX4^+^ female germ cells could be the fact that in females the premeiotic OGON stage is transient and BMP signaling could be downregulated upon meiotic entry. Regarding the localization of pSMAD2, we observed nuclear dots in male and female germ cells and somatic cells in second trimester gonads (Figure 8A; control in Supplementary Figures 3A,B). pSMAD3 showed a similar distribution pattern albeit with lower signal intensity (Figure 8A; control in Supplementary Figures 3A,B).

Our analysis suggested a pronounced activation of the TGFβ/BMP signaling pathway during the transition from PGCs to GONs in both sexes (Figure 8C), validated by nuclear localization of pSMAD1/5/9 and upregulation of *SMAD* and *ID* genes. However, our data suggests that the significant divergence in terms of TGFβ/BMP ligand production between male and female gonadal somatic cells may be most relevant to induce sex-specific somatic differentiation, instead of affecting the transition from PGCs to GONs in a sex-specific manner.

### NOTCH Signaling Ligand–Receptor Interactions Between Germ Cells and Gonadal Somatic Cells

**FIGURE 9:**
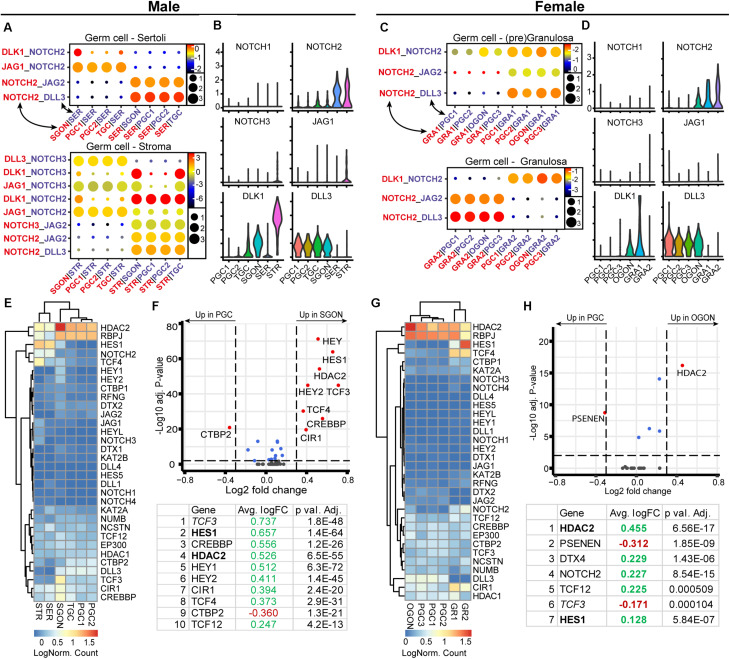
Expression of NOTCH ligand–receptor pairs and downstream pathway effectors in fetal gonads. **(A)** CellphoneDB analysis of NOTCH interactions between different clusters in male fetal gonads. Circle size represents the −log_10_ (*p*-value). Depicted are −log_10_ *p*-values (circle size) and log_2_ means (circle color) for the interacting pairs, for selected pairwise cluster combinations. **(B)**Violin plots of selected NOTCH receptors and ligands in male germ and somatic clusters. **(C)** CellphoneDB analysis of NOTCH interactions between different clusters in female fetal gonads. **(D)** Violin plots of selected NOTCH receptors and ligands in female germ- and somatic clusters. **(E)** Heatmap showing the expression of genes of interest associated with NOTCH signaling pathway in male germ and somatic cell clusters. **(F)** A volcano plot showing NOTCH-related genes between PGC and GON clusters in male gonads. Table shows the top 10 differentially expressed genes. Genes in bold are common between male and female, while genes in italics show divergent regulation. **(G)** Heatmap showing the expression of genes of interest associated with NOTCH signaling pathway in female germ and somatic cell clusters. **(H)** A volcano plot showing NOTCH-related genes between PGC and GON clusters in female gonads. Table shows the top seven differentially expressed genes.

Using CellphoneDB, we observed significant interactions between of NOTCH ligands (*DLK1* and *DLL3*) from germ cells and *NOTCH2* from gonadal somatic cells (Figures 9A–D), in line with a previous analysis (Li et al., 2017). Hence, based on the interactions predicted by CellphoneDB, it seems that germ cells of both sexes may signal toward somatic cells, instead of the other way around (Figures 9A–D). In the view of these results, it was surprising to detect a striking sex-specific upregulation of HEY (*HEY1/2*) and HES (*HES1/5*) transcription factors, which are primary downstream targets of the NOTCH pathway (Vanorny and Mayo, 2017), in SGONs compared with PGCs (Figures 9E–H). In addition, SGONs also showed upregulation of *TCF3*, encoding a binding partner of HES1 (Ikawa et al., 2006; Figures 9E,F).

**FIGURE 10:**
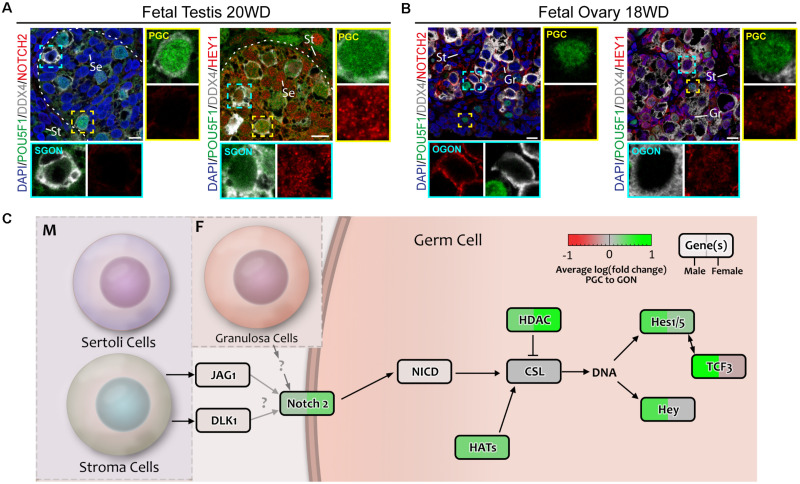
Analysis of NOTCH signaling in fetal gonads by immunostaining for NOTCH2 receptor and downstream effector HEY1. **(A,B)** Immunofluorescence of NOTCH2 and HEY1 with DDX4 and POU5F1 in fetal testis of 20 weeks development (WD) **(A)** and fetal ovary of 18 WD **(B)**. NOTCH2 is absent in the testis, but is highly expressed in female germ cells. HEY1 is present in most cell types of the testis and ovary, suggesting general activation of the NOTCH pathway. Yellow boxes indicate zoom-in of a PGC, and cyan boxes indicate zoom-in of a GON. Se, St, and Gr annotate Sertoli, stromal, and granulosa cells, respectively. Scale bar indicates 10 μm. **(C)** A schematic model of active signaling NOTCH axes in germ cells representing a combination of results from CellphoneDB analysis and differentially expressed gene analysis. Genes upregulated in GONs are depicted in green, while those upregulated in PGCs are depicted in red.

We examined the expression and localization of NOTCH2 (intracellular domain) and HEY1 in fetal gonads by immunofluorescent staining. As expected, NOTCH2 was detected not only in the membrane of gonadal somatic cells, particularly male STR (weak staining) (Figure 10A), but also in female GRA (strong staining) particularly in the vicinity of OGONs (Figure 10B). Although weaker, NOTCH2 was also observed in the gonadal somatic cells in the first trimester, particularly in females (Supplementary Figures 2F,G). This suggested that NOTCH signaling could be taking place in the fetal gonads. Interestingly, the nuclear expression NOTCH target gene HEY1 was ubiquitous in male and female gonads (Figures 10A,B and Supplementary Figures 2F,G), but was particularly strong in the somatic cells including the STR in the second trimester (Figure 10A) and SER in the first trimester (Supplementary Figure 2F), in agreement with active NOTCH signaling in gonads in both sexes (Figure 10C).

## Discussion

An analysis of the transition between human PGCs and GONs and their interaction with the surrounding somatic gonadal tissue has provided a framework to understand the complex spatial and temporal relationships during this developmental phase, considering that sex-specific differentiation has already taken place in the somatic gonads. In mice, *Wnt5A–Ror2* and *Kit–Kitlg* are involved in sustaining the motility of migrating PGCs (Runyan et al., 2006; Gu et al., 2009; Laird et al., 2011). We found that based on receptor–ligand expression analysis, both these interactions occur in human gonadal PGCs and GONs (except *KIT–KITLG* in SGONs), and thus, these are sustained even after PGC migration. WNT5A and KITL signaling may induce motility in germ cells post migration, particularly the inward movement of female germ cells from the cortex to the ovarian cords during the transition from PGCs to GONs. In addition to its role during migration, Kit–Kitlg signaling is essential for germ cell survival and proliferation (Matsui et al., 1991; Pesce et al., 1993), and this could also be the case in human gonadal germ cells (PGCs and GONs). Of interest, both *Wnt5A* and *Ror2*-deficient mutants showed gonads of reduced in size. A delay in in meiosis initiation was reported in the ovaries of *Ror2* mutants, although this may be a downstream consequence of faulty migration (Warr et al., 2009; Arora et al., 2016). Moreover, developmental defects in the testis of *Sfrp1/Sfrp2* double mutants resemble those of *Wnt5a* mutants (Warr et al., 2009). SFRPs are traditionally categorized as secreted inhibitors of canonical WNT signaling (van Loon et al., 2020). Here, we identified *SFRP2* as the highest differentially expressed WNT-related gene during the transition from PGCs to GONs. Whether *SFRP2/WNT5A/ROR2* have a direct role in the transition between PGCs and GONs or also affect germ cell migration remains to be investigated.

In addition to *WNT5A*, we also observed the expression of *WNT2B* and *WNT3* by germ cells (mostly PGCs) and *WNT6* by supporting somatic cells. The role of these particular WNT ligands in mammalian gonad development remains unknown, but activation of canonical WNT may be taking place during the transition from female PGCs to GONs. The current paradigm of mammalian sex determination (in mice) depends on the antagonistic signaling between the Wnt4/Rspo1/Ctnnb1 axis in female gonads and the Sox9/Fgf9 axis in male gonads (Vainio et al., 1999; Chassot et al., 2008; Jameson et al., 2012; Kossack et al., 2019). Considering this, two aspects of our analysis on WNT signaling are striking: no expression of *WNT4* was observed in fetal ovaries and no clear differences exist in WNT ligand expression between the sexes. One possible explanation for this is that *WNT4* is expressed in the ovaries in a brief time window prior to the sample ages of the analyzed dataset. Previous reports on *WNT4* expression in human fetal ovaries are conflicting. Two studies reported no difference in *WNT4* expression between sexes (Tomaselli et al., 2011; Mamsen et al., 2017), while another reported *WNT4* expression throughout development in the ovaries but not the testis (Jääskeläinen et al., 2010). Proof for the role of WNT4/RSPO1 signaling in sex determination in humans relies on rare genetic defects that are the result of mutations in the corresponding genes (Parma et al., 2006; Mandel et al., 2008). Genetically, female *WNT4* or *RSPO1*-deficient individuals show true sex reversal, developing male gonadal tissue. By contrast, gonads of *Wnt4* or *Rspo1* knockout mice show only partial masculinization, whereby ovarian tissue with oocyte nests is still retained (Vainio et al., 1999; Chassot et al., 2008). In fact, germ cells in *WNT4*-deficient ovaries develop normally until E15.5 and progress into early stages of meiosis (Chassot et al., 2008). These species-specific discrepancies suggest that WNT4/RSPO1 signaling may be important in gonadal development at an earlier time point in humans when compared to mice. The fact that *WNT4*-deficient individuals show defects in multiple organs originating from the urogenital ridge supports this notion. Our inability to address these issues experimentally highlights the need for *in vitro* models of human gonadal development.

RTK are large class of cell surface receptors that regulate cell proliferation and differentiation. In addition to *KIT–KITLG*, we identified *TIMP1–FGFR2* and *IGF1–IGFR1* as potentially active RTK signaling axes during the transition from PGCs to GONs. TIMP1 is an inhibitor of metalloproteinases that has been implicated in mammalian follicle maturation and ovulation (Chaffin and Stouffer, 1999; Stouffer et al., 2007; Stilley and Sharpe-Timms, 2012). *Timp1* deficiency in mice results in reduced fertility (Nothnick, 2001) and in *Caenorhabditis elegans* reduces germ cell numbers and results in sterility (Kubota et al., 2019). Moreover, IGF/insulin signaling is an established driver of Sertoli cell proliferation and is required for normal testis development in mice (Nef et al., 2003; Pitetti et al., 2013a, b; Cannarella et al., 2017). Interestingly, the dual conditional knockout of *Insr* and *Igf1r* in DDX4^+^ germ cells (GONs) did not prevent their development through spermatogenesis (Pitetti et al., 2013a); however, the function of *Igf1* (and *Insr*) in the transition from PGCs to GONs in male embryos was not investigated.

Our examination of total ERK (*MAPK1/2*) showed a strong cytoplasmic accumulation specifically in GONs of both sexes. ERK1 and ERK2 are required for oocyte maturation (Su et al., 2003; Fan et al., 2009). Moreover, it has been reported that treatment with RA activates ERK1/2 signaling in mouse OGONs (12 dpf) and that this is required to upregulate RA-induced *Stra8*, a key initiator of meiosis (Kim et al., 2019). The role of ERK activation in *C. elegans* germ cells has also been linked to meiotic progression, inducing phosphorylation of HTP1 (in human HORMAD1) and SYP-2 (in human SYCEs), which controls the assembly and maintenance of the synaptonemal complex (Nadarajan et al., 2016; Das et al., 2020). Should these mechanisms be conserved in humans, we can speculate that the increased expression of ERKs observed in GONs serves to prepare the cells for meiotic entry.

An analysis of the TGFβ/BMP signaling ligands showed a clear divergence between male and female gonads. GRA produced BMP2 and BMP4, while activin A (I*NHBA*) and inhibinB (*INHA* + *INHBB*) were produced by STR and SER, respectively. This fits with the established roles of TGFβ/BMP ligands in mouse gonad development and sex determination. For example, activins/inhibins are important for several aspects of (somatic) testis development (Yao et al., 2006; Archambeault and Yao, 2010; Mendis et al., 2011). Furthermore, activin A/nodal signaling leads to the expression of *NANOS2* in SGONs, preventing their progression into meiosis until adulthood (Souquet et al., 2012; Wu et al., 2013). In female mouse gonads, *Wnt4* is required to induce the expression of *Bmp2* (Yao et al., 2004) and suppress inhibinB (Yao et al., 2006). Knockout of *Bmp2* and *Bmp4* is embryonically lethal early in development and has pronounced effects in PGC specification (Zhang and Bradley, 1996; Lawson et al., 1999; Ying and Zhao, 2001). However, BMP also plays an essential role in the transition from mouse PGC-like cells to DDX4^+^ GON-like cells (Miyauchi et al., 2017). Of note, we observed that *BMP4* is primarily expressed by pre-granulosa cells, while *BMP2* is expressed in granulosa cells, which is in agreement with a previous study on BMP expression in human fetal ovaries (Childs et al., 2010). In male mice, *Bmp8A/B* and *Bmp7* were shown to be important during postnatal spermatogenesis (Zhao et al., 1996, 2001); however, effects during the transition between PGCs and GONs should not be excluded. The presence of pSMAD1/5/9 in DDX4^+^ GONs of both sexes is intriguing. We suggest that the low expression of *BMP4/BMP7* in male germ cells is sufficient to activate the BMP pathway (autocrine) and result in the upregulation of IDs. Alternatively, the observed activation of ERK could also contribute to upregulation of IDs (Zhang, 2017).

Regarding the NOTCH signaling pathway, we observed not only striking *NOTCH2* but also *HEY* and *HES* expression in the somatic compartment of the gonads, as described previously (Li et al., 2017), although HEY1 was also present in PGCs and GONs of both sexes. Considering the lack of *NOTCH2* expression in SGONs, NOTCH-independent pathways may be responsible for *HEY/HES* induction. *HEY* genes can also be induced by BMP/SMAD signaling, even in the absence of functional NOTCH receptors (Zavadil et al., 2004; Sharff et al., 2009; Wöltje et al., 2015). In human embryonic stem cells, *HES1* expression was found to be under the control of BMP and LIF signaling, instead of NOTCH (Kobayashi et al., 2009). In addition, the expression of *HES1* can be induced directly by its binding partner E47 (TCF3) (Ikawa et al., 2006), which we found to be enriched in SGONs. Hence, it is feasible pSMAD1/5/9 activation in GONs drives expression not only of *IDs* but also of *HEY/HES*.

In conclusion, from our molecular analysis of four major signaling pathways WNT, NOTCH, TGFβ/BMP, and RTK, we identified highly expressed signaling ligands in fetal gonads. Of these, *WNT5A*, *WNT6*, *WNT3*, *WNT2B*, and *TIMP1* were expressed in both male and female fetal gonads and may be relevant for *in vitro* germ cell survival and maintenance in general. When considering sex-specific signaling, we suggest that to mimic the transition from female PGCs to OGONs *in vitro* may require activation of the canonical WNT, activation of the BMP pathway (with high levels of BMP2/4), and treatment with KITL. By contrast, the transition from male PGCs to SGONs may benefit from addition of IGF1 and FGF9 and activation of the BMP pathway (with low levels of BMP4/7).

## Data Availability Statement

The datasets presented in this study can be found in online repositories. The names of the repository/repositories and accession number(s) can be found below: GEO database, accession no: GSE86146, available at https://www.ncbi.nlm.nih. gov/geo/query/acc.cgi?acc=GSE86146 and http://github.com/zorrodong/germcell.

## Author Contributions

AO, YC, CR, and JS: data generation. AO, YC, CR, and SC: data analysis and manuscript writing. AO, YC, CR, JS, and SC: approval of the final version. All authors contributed to the article and approved the submitted version.

## Conflict of Interest

The authors declare that the research was conducted in the absence of any commercial or financial relationships that could be construed as a potential conflict of interest.

## Footnotes

1 http://github.com/zorrodong/germcell

2 https://www.genome.jp/kegg/pathway

## Supplementary Material

Supplementary Figure 1 Characterization of different germ cell types in the gonads. **(A)** Immunofluorescence of DDX4, NR2F2, and GATA4 in fetal testis (18 WD) and fetal ovary (19 WD). In testis seminiferous tubes, GATA4^+^ cells are identified as SER, whereas outside the tubules, NR2F2 marks stromal cells (STR). In the fetal ovary, GATA4 marks GRA and NR2F2 marks stromal cells. Dashed lines depict the boundaries of the seminiferous tubes. Se, St, and Gr annotate Sertoli, stromal, and granulosa cells, respectively. Scale bar indicates 10 μm. **(B)** Immunofluorescence of MAGEA3, POU5F1, and DDX4 in fetal testis (12 WD). Three male germ cell types can be discerned based on the following expression pattern: POU5F1^+^/DDX4^Low^/MAGEA3^–^, POU5F1^–^/DDX4^Low^/MAGEA3^–^, and POU5F1^–^/DDX4^*H**i*^/MAGEA3^+^, corresponding to PGCs, TGCs, and SGONs, respectively. Scale bar indicates 10 μm. **(C)** Immunofluorescence of MAGEA3 and DDX4 with NANOG or SOX17 in fetal testis (12 WD). TGCs are identified as NANOG^–^ or SOX17^Low^/DDX4^Low^/MAGEA4^–^ germ cells. Note: SOX17 is highly not only expressed by PGCs but is also expressed at lower levels by TGCs and SGONs, unlike NANOG and POU5F1, which are PGC-exclusive. Scale bar indicates 10 μm. **(D)** Immunofluorescence of MAGEA3, POU5F1, and MKI67 in fetal testis (18 WD). MAGEA3^+^ SGONs are always negative for MKI67, whereas MKI67 staining is observed in POU5F1^+^ PGCs. Scale bar indicates 10 μm.

Supplementary Figure 2 Immunofluorescence for specific signaling pathways. **(A)** Immunofluorescence of phospho (p)-p38 MAPK in fetal testis of 20 WD and fetal ovary of 18 WD, with POU5F1 and DDX4. Yellow boxes indicate zoom-in of a PGC, and cyan boxes indicate zoom-in of a GON. Few nuclear p-p38 MAPK puncta are present in germ cells of either sex, whereas staining intensity is higher in somatic cells, particularly in GRA. Scale bar indicates 10 μm. **(B)** Immunofluorescent staining of total ERK in fetal ovary of 18 WD, with PDPN and DDX4. ERK is enriched in SGONs, and low magnification imaging (scale bar: 5 μm) shows a subcortical population of ERK-enriched germ cells. Scale bar indicates 10 μm. **(C)** Immunofluorescence of MAPK signal effectors in the first trimester 10 WD fetal testis. p-p38 MAPK, pJNK, pERK, or total ERK were stained, with POU5F1 and DDX4. PGCs were negative for these markers. Scale bar indicates 10 μm. **(D)** Immunofluorescence of MAPK signal effectors in the first trimester 9 or 10 WD fetal ovary. Similar to male PGCs, female first trimester PGCs were negative for MAPK signal effectors, except for total ERK, which was observed in DDX4^+^ OGONs. In addition, a strong pERK signal was present in somatic cells surrounding the PGCs. Scale bar indicates 10 μm. **(E)** Immunofluorescence of SFRP2 18-WD fetal ovary with PDPN and DDX4. SFRP2 is expressed in PGCs, and low magnification imaging reveals SFRP2 enrichment near the cortex. Scale bar indicates 10 μm in right panels (high magnification) and 50 μm in left panels (low magnification). **(F)** Immunofluorescence of NOTCH2 or HEY1 in the first trimester 10-WD fetal testis and 9-WD fetal ovary, with POU5F1 and DDX4. NOTCH2 was not present in the testis but was expressed in female germ cells, albeit with lower intensity. HEY1 was present in the ovary in most cells. In males, HEY1 was more specifically expressed in somatic cells and largely absent from germ cells. Scale bar indicates 10 μm.

Supplementary Figure 3 Positive controls for antibodies recognizing phosphorylated proteins. **(A)** Positive control experiment for antibodies recognizing phosphorylated proteins. HEK293T cells, cultured with 10% FCS (left panels), were serum starved for 16 h (middle panels), treated for 30 min with the indicated growth factors (right panels), and immunostained for the respective antibodies (pERK, pSAPK/JNK, p-p38MAPK, pSMAD1/5/9, pSMAD2, and pSMAD3). Scale bars indicate 50 μm. **(B)** Positive control staining for pSMAD2 and pSMAD3 antibodies on fetal intestine. Nuclear pSMAD2 and pSmad3 were observed in intestinal endoderm. Scale bars indicate 50 μm.
