# Supplementary figures and images for: Ligand–Receptor Interactions Elucidate Sex-Specific Pathways in the Trajectory From Primordial Germ Cells to Gonia During Human Development
Source: Front Cell Dev Biol. 2021 Jun 18;9:661243. doi: 10.3389/fcell.2021.661243 (PMC8253161; doi:10.3389/fcell.2021.661243)

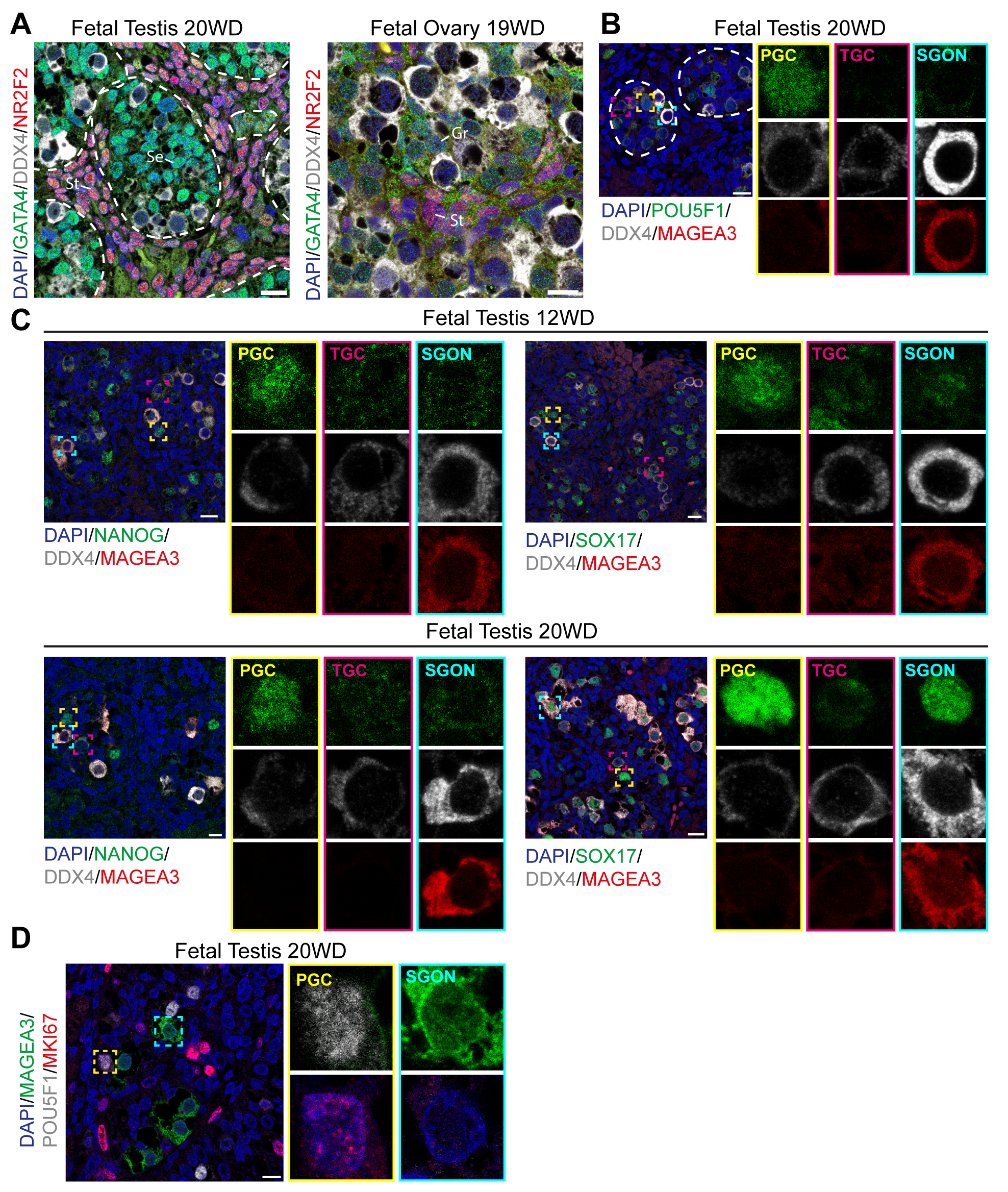

Supplement: Supplementary Figure 1 — Characterization of different germ cell types in the gonads. (A) Immunofluorescence of DDX4, NR2F2, and GATA4 in fetal testis (18 WD) and fetal ovary (19 WD). In testis seminiferous tubes, GATA4+ cells are identified as SER, whereas outside the tubules, NR2F2 marks stromal cells (STR). In the fetal ovary, GATA4 marks GRA and NR2F2 marks stromal cells. Dashed lines depict the boundaries of the seminiferous tubes. Se, St, and Gr annotate Sertoli, stromal, and granulosa cells, respectively. Scale bar indicates 10 μm. (B) Immunofluorescence of MAGEA3, POU5F1, and DDX4 in fetal testis (12 WD). Three male germ cell types can be discerned based on the following expression pattern: POU5F1+/DDX4Low/MAGEA3–, POU5F1–/DDX4Low/MAGEA3–, and POU5F1–/DDX4Hi/MAGEA3+, corresponding to PGCs, TGCs, and SGONs, respectively. Scale bar indicates 10 μm. (C) Immunofluorescence of MAGEA3 and DDX4 with NANOG or SOX17 in fetal testis (12 WD). TGCs are identified as NANOG– or SOX17Low/DDX4Low/MAGEA4– germ cells. Note: SOX17 is highly not only expressed by PGCs but is also expressed at lower levels by TGCs and SGONs, unlike NANOG and POU5F1, which are PGC-exclusive. Scale bar indicates 10 μm. (D) Immunofluorescence of MAGEA3, POU5F1, and MKI67 in fetal testis (18 WD). MAGEA3+ SGONs are always negative for MKI67, whereas MKI67 staining is observed in POU5F1+ PGCs. Scale bar indicates 10 μm. [file Image_1.TIF]

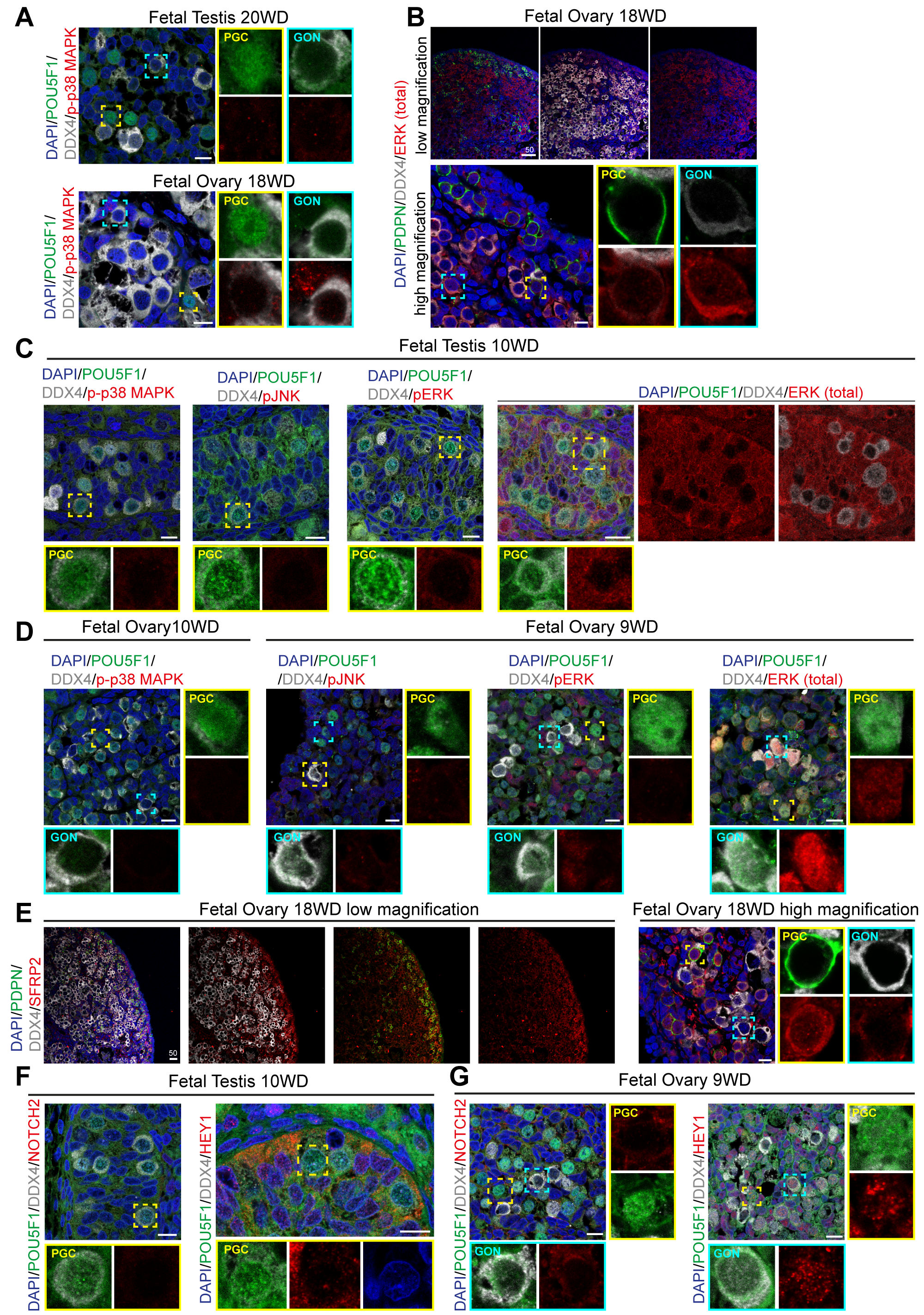

Supplement: Supplementary Figure 2 — Immunofluorescence for specific signaling pathways. (A) Immunofluorescence of phospho (p)-p38 MAPK in fetal testis of 20 WD and fetal ovary of 18 WD, with POU5F1 and DDX4. Yellow boxes indicate zoom-in of a PGC, and cyan boxes indicate zoom-in of a GON. Few nuclear p-p38 MAPK puncta are present in germ cells of either sex, whereas staining intensity is higher in somatic cells, particularly in GRA. Scale bar indicates 10 μm. (B) Immunofluorescent staining of total ERK in fetal ovary of 18 WD, with PDPN and DDX4. ERK is enriched in SGONs, and low magnification imaging (scale bar: 5 μm) shows a subcortical population of ERK-enriched germ cells. Scale bar indicates 10 μm. (C) Immunofluorescence of MAPK signal effectors in the first trimester 10 WD fetal testis. p-p38 MAPK, pJNK, pERK, or total ERK were stained, with POU5F1 and DDX4. PGCs were negative for these markers. Scale bar indicates 10 μm. (D) Immunofluorescence of MAPK signal effectors in the first trimester 9 or 10 WD fetal ovary. Similar to male PGCs, female first trimester PGCs were negative for MAPK signal effectors, except for total ERK, which was observed in DDX4+ OGONs. In addition, a strong pERK signal was present in somatic cells surrounding the PGCs. Scale bar indicates 10 μm. (E) Immunofluorescence of SFRP2 18-WD fetal ovary with PDPN and DDX4. SFRP2 is expressed in PGCs, and low magnification imaging reveals SFRP2 enrichment near the cortex. Scale bar indicates 10 μm in right panels (high magnification) and 50 μm in left panels (low magnification). (F) Immunofluorescence of NOTCH2 or HEY1 in the first trimester 10-WD fetal testis and 9-WD fetal ovary, with POU5F1 and DDX4. NOTCH2 was not present in the testis but was expressed in female germ cells, albeit with lower intensity. HEY1 was present in the ovary in most cells. In males, HEY1 was more specifically expressed in somatic cells and largely absent from germ cells. Scale bar indicates 10 μm. [file Image_2.TIF]

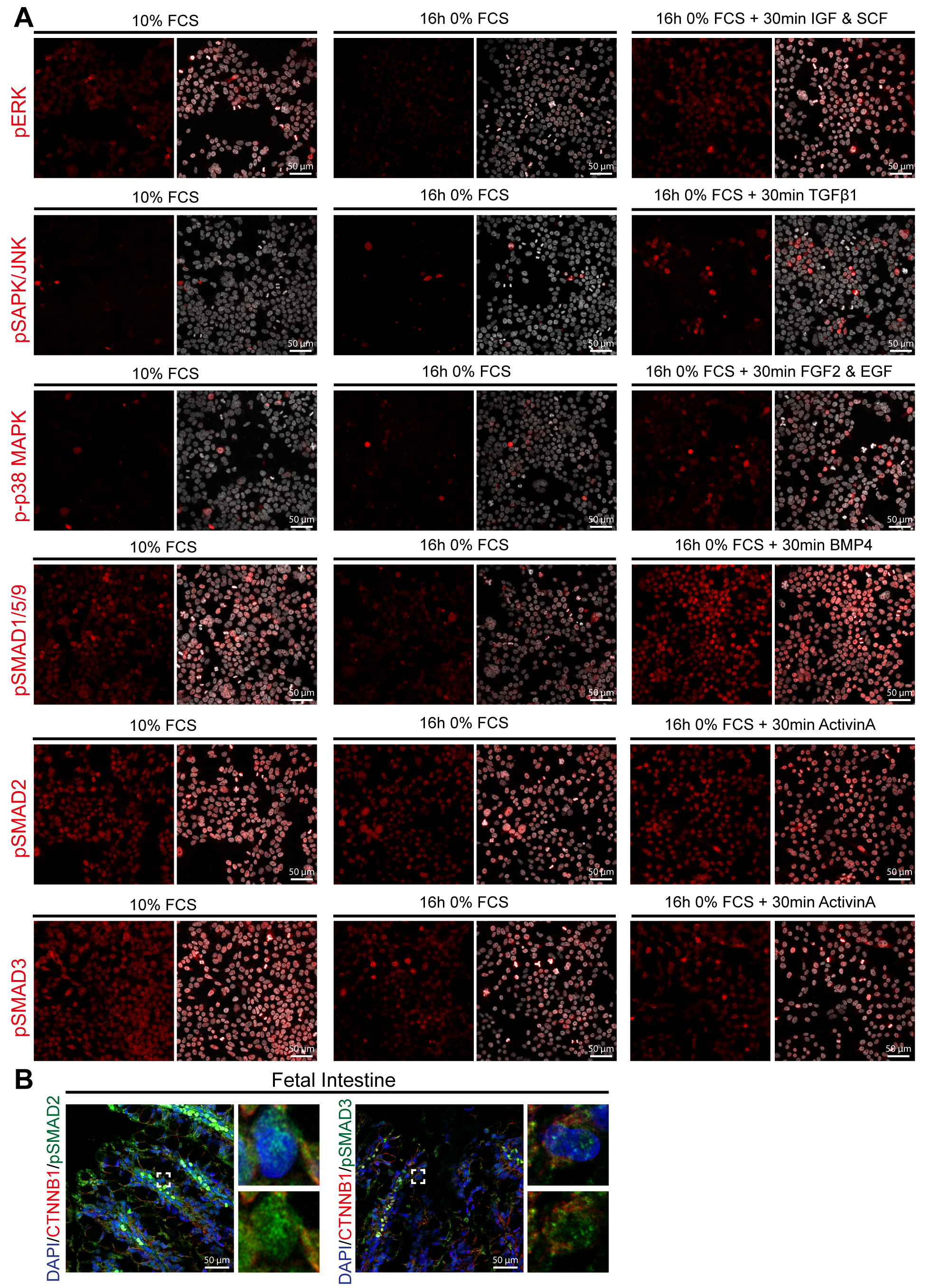

Supplement: Supplementary Figure 3 — Positive controls for antibodies recognizing phosphorylated proteins. (A) Positive control experiment for antibodies recognizing phosphorylated proteins. HEK293T cells, cultured with 10% FCS (left panels), were serum starved for 16 h (middle panels), treated for 30 min with the indicated growth factors (right panels), and immunostained for the respective antibodies (pERK, pSAPK/JNK, p-p38MAPK, pSMAD1/5/9, pSMAD2, and pSMAD3). Scale bars indicate 50 μm. (B) Positive control staining for pSMAD2 and pSMAD3 antibodies on fetal intestine. Nuclear pSMAD2 and pSmad3 were observed in intestinal endoderm. Scale bars indicate 50 μm. [file Image_3.TIF]
