# Supplementary material for: Ligand–Receptor Interactions Elucidate Sex-Specific Pathways in the Trajectory From Primordial Germ Cells to Gonia During Human Development
Source: Front Cell Dev Biol. 2021 Jun 18;9:661243. doi: 10.3389/fcell.2021.661243 (PMC8253161; doi:10.3389/fcell.2021.661243)
Supplement: Supplementary file 4 [file Table_1.docx]

| **Primary Antibodies** | | | | |
| --- | --- | --- | --- | --- |
| **Target** | **Catalogue #** | **Manufacturer** | **Species** | **Dilution** |
| β-catenin | 610154 | BD Biosciences | Mouse | 1:500 |
| CKIT | A450229-2 | DAKO | Rabbit | 1:500 |
| DDX4 (VASA) | AF2030 | R&D systems | Goat | 1:500 |
| DDX4 (VASA) | ab13840 | Abcam | Rabbit | 1:500 |
| HEY1 | ab22614 | Abcam | Rabbit | 1:200 |
| Notch2 | # 5732 | Cell Signaling | Rabbit | 1:200 |
| p44/42 MAPK (ERK1/2) | #9102 | Cell Signaling | Rabbit | 1:200 |
| phospho-JNK | #4668 | Cell Signaling | Rabbit | 1:200 |
| phospho-p38 MAPK | #4631 | Cell Signaling | Rabbit | 1:200 |
| phospho-p44/42 MAPK (ERK1/2) | #9101 | Cell Signaling | Rabbit | 1:200 |
| phospho-Smad1/5/9 | #13820 | Cell Signaling | Rabbit | 1:200 |
| Podoplanin | ab77854 | Abcam | Mouse | 1:500 |
| POU5F1 (Oct4) | sc-5279 | Santa Cruz | Mouse | 1:100 |
| SFRP2 | PA5-29390 | Thermo Fisher | Rabbit | 1:200 |
| SOX17 | AF1924 | R&D systems | Goat | 1:500 |
| TCF7L2 | MA5-35295 | Thermo Fisher | Rabbit | 1:200 |
| Phospho-SMAD2 | #3108 | Cell Signaling | Rabbit | 1:200 |
| Phospho-SMAD3 | #9520 | Cell Signaling | Rabbit | 1:200 |
| COUP-TFII | #6434 | Cell Signaling | Rabbit | 1:200 |
| GATA4 | sc-25310 | Santa Cruz | Mouse | 1:200 |
| MAGEA3 | MABC1150 | Millipore | Mouse | 1:200 |
| KI67 | ab15580 | Abcam | Rabbit | 1:200 |
| NANOG | AF1997 | R&D systems | Goat | 1:200 |
| **Secondary Antibodies** | | | | |
| Donkey anti-Mouse IgG (H+L) Highly Cross-Adsorbed Secondary Antibody, Alexa Fluor 488 | A-21202 | Thermo Fisher | Donkey | 1:500 |
| Donkey anti-Rabbit IgG (H+L) Highly Cross-Adsorbed Secondary Antibody, Alexa Fluor 555 | A-31572 | Thermo Fisher | Donkey | 1:500 |
| Donkey anti-Goat IgG (H+L) Cross-Adsorbed Secondary Antibody, Alexa Fluor 647 | A-21447 | Thermo Fisher | Donkey | 1:500 |

**Supplementary Table 1: List of antibodies used for immunostaining**
